# Cajanolactone A, a Stilbenoid From *Cajanus canjan* (L.) Millsp, Prevents High-Fat Diet-Induced Obesity via Suppressing Energy Intake

**DOI:** 10.3389/fphar.2021.695561

**Published:** 2021-05-31

**Authors:** Zhuohui Luo, Jiawen Huang, Zhiping Li, Zhiwen Liu, Linchun Fu, Yingjie Hu, Xiaoling Shen

**Affiliations:** Science and Technology Innovation Center, Guangzhou University of Chinese Medicine, Guangzhou, China

**Keywords:** cajanolactone A, high-fat diet, obesity, adipose tissue, liver, energy intake

## Abstract

Cajanolactone A (CLA) is a stilbenoid isolated from *Cajanus canjan* (L.) Millsp with the potential to prevent postmenopausal obesity. In this study, the effect of CLA on high-fat diet (HFD)-induced obesity in female C57BL/6 mice was investigated. It was found that, treatment with CLA reduced the energy intake and effectively protected the mice from HFD-induced body weight gain, fat accumulation within the adipose tissues and liver, and impairment in energy metabolism. Further investigation revealed that CLA significantly down-regulated the expression of *ORX*, *ORXR2*, *pMCH*, and *Gal* in the hypothalamus and antagonized HFD-induced changes in the expression of UCP1, *Pgc-1*α, *Tfam*, and *Mfn1* in the inguinal white adipose tissue (iWAT); Caveolin-1, MT and UCP3 in the perigonadal white adipose tissue (pWAT); and *Pdhb*, *IRS2*, *Mttp*, *Hadhb*, and *Cpt1b* in the liver. CLA also protected the pWAT and liver from HFD-induced mitochondrial damage. However, neither HFD nor CLA showed an effect on the mass of brown adipose tissue (BAT) or the expression of UCP1 in the BAT. In summary, our findings suggest that CLA is a potential drug candidate for preventing diet-induced obesity, at least in females. CLA works most likely by suppressing the hypothalamic expression of orexigenic genes, which leads to reduced energy intake, and subsequently, reduced fat accumulation, thereby protecting the adipose tissues and the liver from lipid-induced mitochondrial dysfunction.

## Introduction

Obesity is one of the most dangerous factors in triggering insulin resistance, non-alcoholic fatty liver disease, type II diabetes, etc. (Reaven, 2002; Després and Lemieux, 2006; Meldrum et al., 2017). As obesity is the result of an imbalance between long-term energy intake and consumption, reducing energy intake and/or dissipating excess energy intake are feasible strategies to prevent and ameliorate obesity (Crisan et al., 2008; Elabd et al., 2009).

To reduce energy intake, the safest method is to eat less. However, long-term diet control is difficult to achieve for people who love good food, especially in a society where food is plentiful. Therefore, medications such as Liraglutide (Lin et al., 2020) and Qsymia (Cohen and Gadde, 2019), which suppress the appetite, have roles to play in people suffering from overweight. On the other hand, preventing the absorption of high-calorie food in the gastrointestinal tract is an effective way to reduce energy intake and storage, which can be accomplished via Orlistat. Orlistat inactivates the lipases in the gut by binding to them to prevent the lipase-catalyzed hydrolysis of triglycerides and the consequent absorption of the hydrolysates (free fatty acids and monoglycerides) (Curran and Scott, 2004).

To increase energy expenditure, engaging in more exercise is always the primary advice doctors give to their patients. However, exercise is often challenging for overweight and obese people to engage in long-term and is not suitable for all patients. Therefore, strategies promoting the basal metabolic rate—for example, stimulating uncoupling protein 1 (UCP1)-dependent heat production via the brown or beige adipose tissues or promoting aerobic metabolism in the mitochondria of functional tissues—are needed (Kim and Plutzky, 2016).

Adipose plays an important role in energy storage and energy expenditure (Sun et al., 2011). White adipose tissue (WAT) and brown adipose tissue (BAT) are two general types of adipose tissue in mammals. WAT stores energy as fat in the adipocytes, whereas BAT, which specifically expresses UCP1 in the brown adipocytes, consumes fat to produce heat and expends more energy than WAT and other tissues. The way that WAT expands and remodels directly influences metabolic syndrome in the obesity process (Vishvanath and Gupta, 2019). Meanwhile, BAT activity is reported to be impaired in patients with obesity, suggesting an association between BAT activity and obesity (Villarroya et al., 2017). Recent studies revealed that BAT and beige adipocytes could be promising therapeutic targets for metabolic disease (Contreras et al., 2016). Beige adipocytes, which exist in subcutaneous WAT, can also produce heat by expressing UCP1 (Barbatelli et al., 2010; Cinti, 2011; Montanari et al., 2017), a process called non-shivering thermogenesis (Cannon and Nedergaard, 2004; Giralt et al., 2013). It is a novel approach to explore the effect of drug on ameliorating obesity from the perspective of promoting WAT browning.

In eukaryotes, mitochondrial function is essential for the transformation of nutrients absorbed or energy stored (fat) into heat through UCPs, or ATP through oxidative phosphorylation (Villarroya et al., 2009). Studies have reported that the impairment of the biogenesis and the function of the mitochondria is closely related to obesity (Rong et al., 2007; Yin et al., 2014). Mitochondrial function in adipose tissue has been regarded as a key target for obesity (Medina-Gomez, 2012). Thus, the biochemical indicators related to mitochondrial biogenesis, as well as their functions, are another focus of this study.

Cajanolactone A (CLA) is a stilbenoid discovered by us from the leaves of *Cajanus canjan* (L.) Millsp promoting osteoblast differentiation in human bone marrow mesenchymal stem cells (Liu et al., 2019). Recently, CLA has been proven to have potential use in treating postmenopausal obesity by protecting the ovariectomized mice from obesity and liver steatosis (Luo et al., 2020; Luo et al., 2021). Because CLA prevents estrogen deficiency-induced obesity in a non-estrogen-like manner, it is suggested that CLA may also have the potential to prevent diet-induced obesity in premenopausal women. Therefore, in this study, we investigated the effects of CLA on high-fat diet (HFD)-induced obesity and liver steatosis in female C57BL/6 mice with normal ovarian function. The effects of CLA on the feeding behavior, whole-body energy metabolism, BAT function, WAT browning and mitochondrial function of functional tissues were also investigated.

## Materials and Methods

### Materials and Chemicals

CLA was obtained from *Cajanus canjan* (L.) Millsp at a purity of 98% (assessed by HPLC), as previously reported (Liu et al., 2019). All other chemicals were purchased from Sigma-Aldrich Co. unless otherwise specified.

### Animals

Female C57BL/6 mice at 8 w of age were purchased from Guangdong Medical Laboratory Animal Center (No.44007200061828). The animals were housed in a specific pathogen free environment, with a 12 h/12 h light/dark cycle, controlled temperature, free water, and chow food. Animals were divided into three groups randomly with eight mice in each group. Animals in the normal control group (NC) were fed a rodent maintenance feed, while animals in the model group (HFD) and CLA-treatment group (CLA) were fed a HFD (60% kcal from fat; D12492; Research diets, Guangdong Medical Laboratory Animal Center, Guangdong, China) for 16 weeks. Mice in the CLA group were treated with 40 mg/kg/day of CLA—the highest dosage used previously (Luo et al., 2020), while in the NC group and HFD group received a vehicle by gavage once daily for 16 weeks. All animal experimental procedures were approved by the Ethics Committee of Guangzhou University of Chinese Medicine (No.20190321007). Animals were weighed once a week.

### Metabolism Monitoring

At the end of the 16th week, mice were placed individually in metabolic measurement cages equipped with a Promethion monitoring system (SABLE) for 24 h adaption. Then, the energy expenditure of the mice was measured via indirect calorimetry over the next 48 h. Energy intake, heat production, respiratory exchange ratio (RER), carbon dioxide production (VCO_2_), oxygen consumption (VO_2_) and *X*-axis ambulatory (XAMB) values were recorded as indicators.

### Body Composition Analysis

After metabolism monitoring, all mice were anesthetized by intraperitoneal injection of sodium pentobarbital (50 mg/kg). The body BAT and WAT were scanned by a Latheta LCT-200 Micro CT (Hitachi Aloka, Tokyo, Japan). Fat mass, fat ratio, visceral fat mass, subcutaneous fat mass and BAT mass were calculated using the Latheta V3.51 software. Three-dimensional reconstruction images of BAT were also constructed.

### Immunofluorescence Analysis

Perigonadal white adipose tissues (pWATs) were isolated (about 0.5 cm^3^) and immersed in 1% PFA for 1 h and then washed by PBS. For LipidTOX imaging, tissues were stained by HCS LipidTOX™ Deep Red Neutral Lipid Stain (1:500, H34477, Thermo Fisher Scientific) for 30 min, images were obtained on an inverted Leica TCS SP8 confocal microscope (Leica, Germany), and 3D images were composited by taking z-stack images using LAS X software-determined levels along the vertical axis. For PECAM-1 imaging, tissues were sealed using 1% goat serum (SL038, Solarbio LIFE SCIENCES, Beijing, China) for 1 h and then incubated with PECAM-1 antibody (1:1,000, MAB1398Z, Millipore, United States) overnight at 4°C. Tissues were washed and incubated with Goat Anti-Armenian hamster IgG H&L (Alexa Fluor® 647) (1:1,000, ab173004, Abcam, United States) for 1 h, images were obtained on an inverted confocal microscope and 3D images were again composited using the LAS X software.

### Hematoxylin-Eosin Staining and Oil Red O Staining

For histopathological analysis, tissues were fixed in 4% paraformaldehyde for 48 h, embedded in paraffin wax, and cut into 5 μm sections. Paraffin sections were stained using hematoxylin and eosin (H and E). For detection of triglyceride deposition, livers embedded in the optimum cutting temperature compound were cryosectioned into 7 μm-thick sections and stained using an oil red O staining solution. Images (× 400) of each slide from non-overlapping fields were taken randomly using an Olympus IX73 microscope (Olympus Corporation, Japan).

### Transmission Electron Microscopy

Fresh pWATs and liver tissues were cut into small pieces and fixed in 2.5% glutaraldehyde (pH 7.4), post-fixed in buffered osmium tetroxide, dehydrated in ethanol, embedded in embed-812 resin, and then polymerized at 60°C for 48 h. Ultra-thin sections were obtained using a Leica EMUC6 ultramicrotome and stained with uranyl acetate and lead citrate. Images were captured with a HT7700 transmission electron microscope (HITACHI, Japan).

### Real-Time Quantitative Polymerase Chain Reaction (RT-qPCR)

The total RNA of the fresh tissues (hypothalami, inguinal white adipose tissue (iWATs) and livers) was individually extracted using a Trizol reagent (Life technologies, MD, United States) and was reverse transcribed to cDNA using a RT-qPCR kit (Invitrogen, CA, United States). The mRNA levels of target genes were quantified in an ABI 7500 Real-Time PCR System (Applied Biosystems, CA, United States) using a SYBR Green qPCR master mix (Takara Biomedical Technology Co.Ltd., Dalian, China). The primer sequences used are shown in Table 1.

**TABLE 1:** Sequences of PCR primers.

Gene name	GenBank	Forward primer (5′ → 3′)	Reverse primer (5′ → 3′)
*MCP-1*	NM_011333	TTA​AAA​ACC​TGG​ATC​GGA​ACC​AA	GCA​TTA​GCT​TCA​GAT​TTA​CGG​GT
*Emr1*	NM_010130	CCC​CAG​TGT​CCT​TAC​AGA​GTG	GTG​CCC​AGA​GTG​GAT​GTC​T
*Apob*	NM_009693	TTG​GCA​AAC​TGC​ATA​GCA​TCC	TCA​AAT​TGG​GAC​TCT​CCT​TTA​GC
*Mttp*	NM_008642	CTC​TTG​GCA​GTG​CTT​TTT​CTC​T	GAG​CTT​GTA​TAG​CCG​CTC​ATT
*Scarb1*	NM_016741	TTT​GGA​GTG​GTA​GTA​AAA​AGG​GC	TGA​CAT​CAG​GGA​CTC​AGA​GTA​G
*Lrp1*	NM_008512	ACT​ATG​GAT​GCC​CCT​AAA​ACT​TG	GCA​ATC​TCT​TTC​ACC​GTC​ACA
*Pdhb*	NM_024221	AGG​AGG​GAA​TTG​AAT​GTG​AGG​T	ACT​GGC​TTC​TAT​GGC​TTC​GAT
*Cpt1a*	NM_013495	CTC​CGC​CTG​AGC​CAT​GAA​G	CAC​CAG​TGA​TGA​TGC​CAT​TCT
*Cpt1b*	NM_009948	GCA​CAC​CAG​GCA​GTA​GCT​TT	CAG​GAG​TTG​ATT​CCA​GAC​AGG​TA
*Cpt1c*	NM_153679	TCT​TCA​CTG​AGT​TCC​GAT​GGG	ACG​CCA​GAG​ATG​CCT​TTT​CC
*Hadha*	NM_178878	TGC​ATT​TGC​CGC​AGC​TTT​AC	GTT​GGC​CCA​GAT​TTC​GTT​CA
*Hadhb*	NM_145558	ACT​ACA​TCA​AAA​TGG​GCT​CTC​AG	AGC​AGA​AAT​GGA​ATG​CGG​ACC
*IRS2*	NM_001081212	CTG​CGT​CCT​CTC​CCA​AAG​TG	GGG​GTC​ATG​GGC​ATG​TAG​C
*Pgc-1α*	NM_008904	TAT​GGA​GTG​ACA​TAG​AGT​GTG​CT	CCA​CTT​CAA​TCC​ACC​CAG​AAA​G
*NPY*	NM_023456	ATG​CTA​GGT​AAC​AAG​CGA​ATG​G	TGT​CGC​AGA​GCG​GAG​TAG​TAT
*Gal*	NM_010253	GGC​AGC​GTT​ATC​CTG​CTA​GG	CTG​TTC​AGG​GTC​CAA​CCT​CT
*pMCH*	NP_084247	GTC​TGG​CTG​TAA​AAC​CTT​ACC​TC	CCT​GAG​CAT​GTC​AAA​ATC​TCT​CC
*ORX*	NM_010410	GTC​GCC​AGA​AGA​CGT​GTT​C	GGT​GGT​AGT​TAC​GGT​CGG​AC
*ORXR2*	NM_198962	GAG​GAT​TCC​CTC​TCT​CGT​CG	GGT​GTA​GGT​ATT​CCC​TCC​ACA
*CARTPT*	NP_038760	CCC​GAG​CCC​TGG​ACA​TCT​A	GCT​TCG​ATC​TGC​AAC​ATA​GCG
*Tfam*	NM_009360	ATT​CCG​AAG​TGT​TTT​TCC​AGC​A	TCT​GAA​AGT​TTT​GCA​TCT​GGG​T
*Mfn1*	NM_024200	CCT​ACT​GCT​CCT​TCT​AAC​CCA	AGG​GAC​GCC​AAT​CCT​GTG​A
*Mfn2*	NM_133201	CAT​TCT​TGT​GGT​CGG​AGG​AG	AAG​GAG​AGG​GCG​ATG​AGT​CT
*Bip*	NM_001163434	ACT​TGG​GGA​CCA​CCT​ATT​CCT	ATC​GCC​AAT​CAG​ACG​CTC​C
β*-actin*	NM_007393	GGC​TGT​ATT​CCC​CTC​CAT​CG	CCA​GTT​GGT​AAC​AAT​GCC​ATG​T

### Western Blot Analysis

Fresh tissues were lysed using a RIPA lysis buffer containing both PMSF and phosphatase inhibitors. An enhanced BCA protein assay kit was used for protein quantification. Samples were mixed with a loading buffer (5 ×), heated for 5 min at 95–100°C and stored at −20°C. SDS-PAGE gels were used to isolate the proteins, and then the proteins were transferred to PVDF membranes. Membranes were sealed with 5% nonfat milk and then incubated overnight at 4°C with relative primary antibodies (Table 2). Then, the anti-rabbit secondary antibody (ab6721, 1:10,000, Abcam, United States) was incubated with the membranes for 1 h and finally combined with ECL reagent (Millipore Corporation, Billerica, United States) for a color reaction.

**TABLE 2:** Primary antibodies.

Name	Host	No	Ratio	Source
UCP1	Rabbit	ab209483	1:2,000	Abcam
Caveolin-1	Rabbit	ab32577	1:2,000	Abcam
MT	Rabbit	ab235036	1:2,000	Abcam
UCP3	Rabbit	NBP2-24608	1:2,000	Novus
β-actin	Rabbit	YT0099	1:10,000	ImmunoWay

### Statistical Analysis

The values were expressed as the mean ± standard error of the mean (SEM). The significance of the differences was analyzed using a one-way ANOVA followed by Bonferroni’s post hoc test. GraphPad Prism 6.0 (San Diego, CA, United States) was used for statistical analysis. *p* values of 0.05 or less were considered significant.

## Results

### CLA Prevented HFD-Induced Body Weight Gain and Fat Accumulation in Mice With Normal Ovarian Function

**FIGURE 1:**
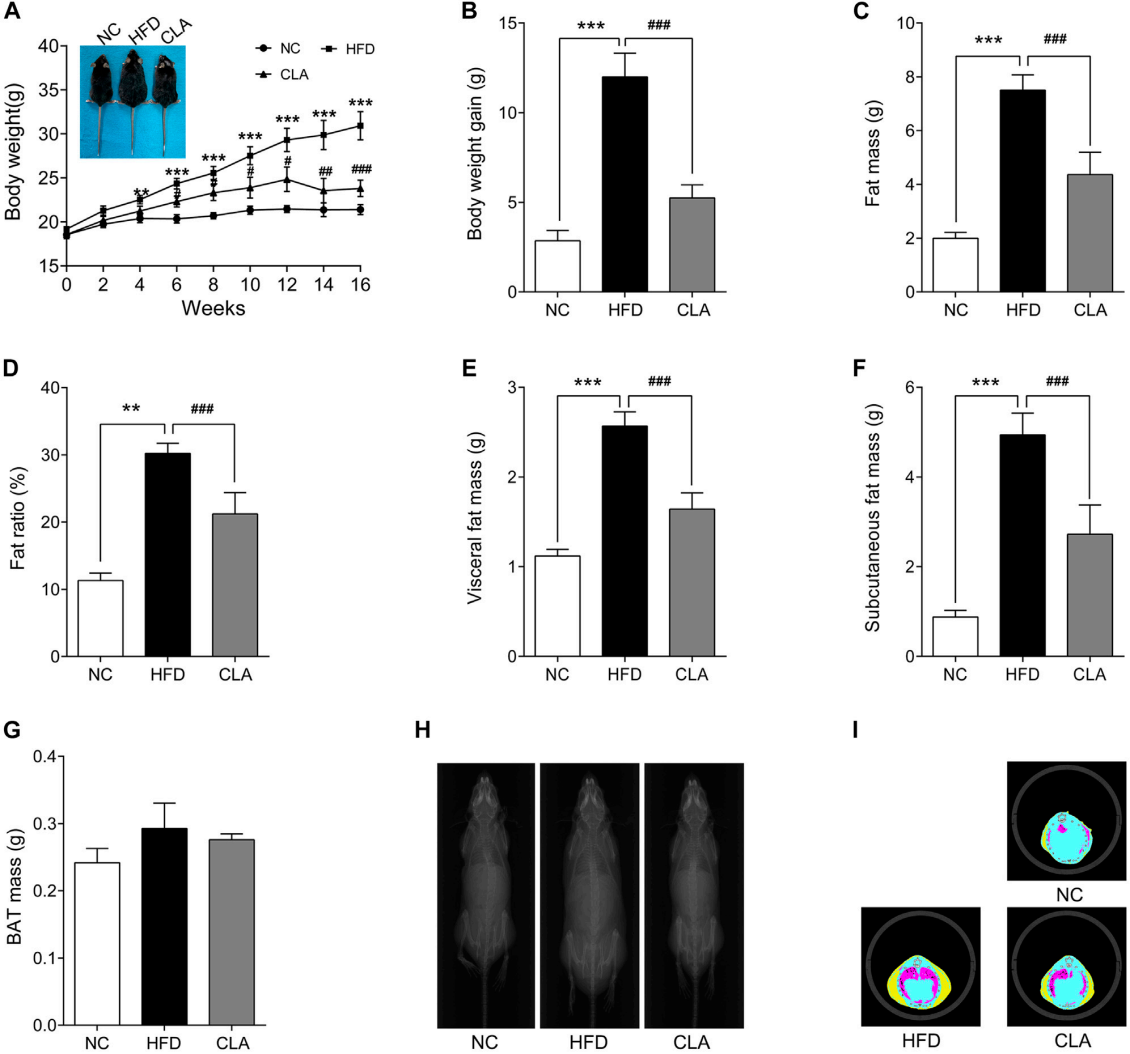
Effect of cajanolactone A (CLA) on high-fat-diet (HFD)-induced obesity in female C57BJ/6 mice. Mice were randomly divided into three groups and fed a regular diet (NC group), high-fat diet (HFD group), or high-fat diet combined with CLA treatment (CLA group) for 16 weeks. Body weights **(A)** were recorded biweekly, and body weight gains **(B)** were calculated at the end of the experiment. Body composition, including the fat mass of the whole body **(C)**, fat ratio **(D)** (Fat ratio % = Fat mass/body weight × 100%), visceral fat mass **(E)**, subcutaneous fat mass **(F)** and BAT mass **(G)**, X-ray portraits of the mice **(H)**, and tomographic images of the mice **(I)** (yellow: subcutaneous fat; red: visceral fat; blue: lean) were obtained on a Latheta LCT-200 Micro CT (Hitachi Aloka, Tokyo, Japan). Data are shown as the mean ± SEM (n = 8). **p < 0.01, ***p < 0.001 vs. NC. ^*#*^ *p* < 0.05, ^*##*^ *p* < 0.01, ^*###*^ *p* < 0.001 vs. HFD.

In this study, mice in the HFD group gained weights rapidly during the 16 w of the experiment (Figures 1A,B), analysis of the body composition revealed excessively accumulated visceral and subcutaneous fat (Figures 1C–F **,** 1H–I) and significantly increased body fat ratio (Figure 1D). Compared to mice in the HFD group, mice in the CLA group grew obviously slower (Figures 1A,B) and accumulated less visceral and subcutaneous fat (Figures 1C–F **,** 1H–I), indicating that treatment with CLA effectively prevented HFD-induced weight gain and development of obesity in female mice with normal ovarian function, may have the potential to prevent premenopausal obesity induced by hyperphagia. In this experiment, HFD and CLA yielded no significant changes in BAT mass, both when assessed by the absolute mass (g) (Figure 1G) and when assessed by the mass relative to the body weight (g/g) (data not shown).

### CLA Protected the Aerobic Metabolic Rate and Reduced the Energy Intake in Female Mice Fed a HFD

**FIGURE 2:**
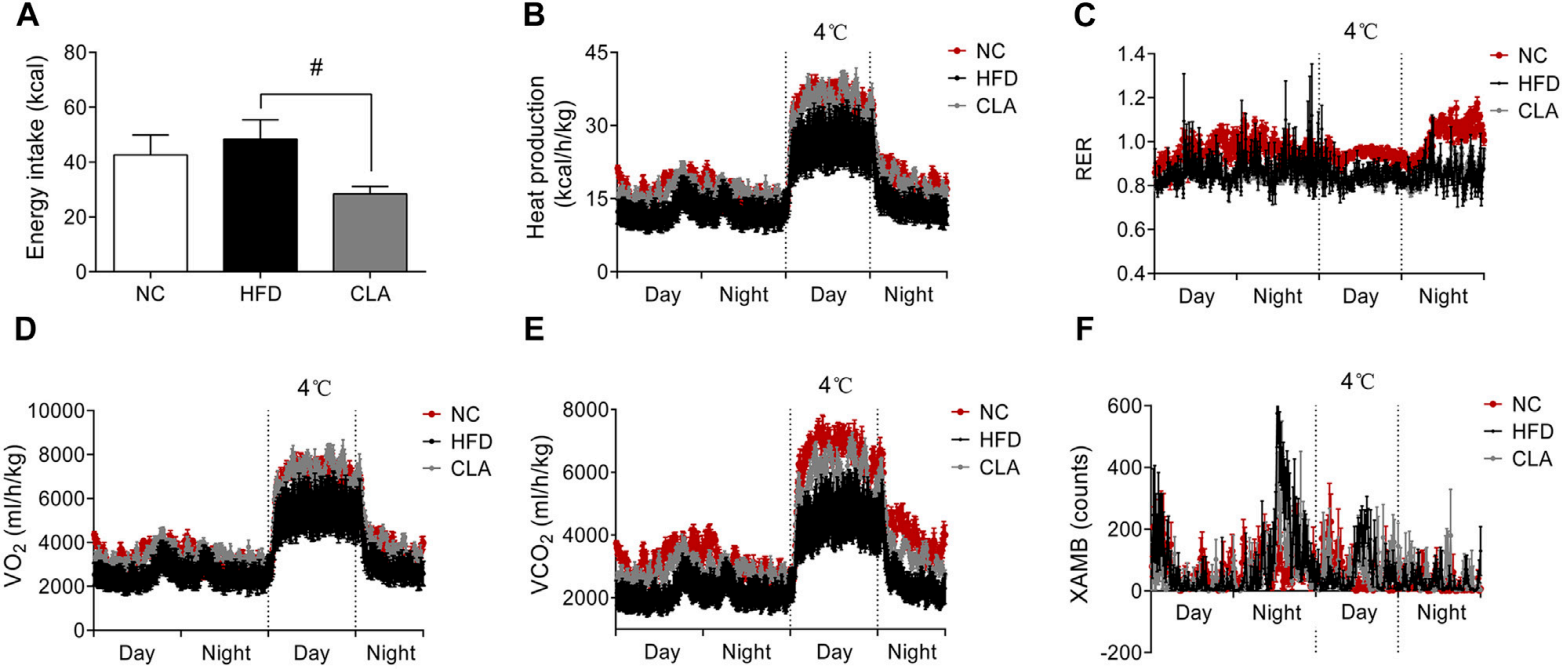
Effects of CLA on the whole-body energy metabolism of mice given a HFD for 16w. Mice were caged individually for 48 h the energy intake **(A)**, heat production **(B)**, respiratory exchange rate (RER) **(C)**, oxygen production (VO_2_) **(D)**, carbon dioxide production (VCO_2_) **(E)**, and *X*-axis ambulatory (XAMB) value **(F)** of the mice were monitored by a Promethion metabolic cage system (Sable Systems, Las Vegas, NV). Data are shown as the mean ± SEM (*n* = 5). ^*#*^ *p* < 0.05, vs. HFD.

To investigate whether CLA prevents obesity through promoting energy metabolism or through suppressing energy intake, a 48 h monitoring of whole body energy expenditure and energy intake was performed on the three groups of mice, and the results are shown in Figure 2. Compared to the mice fed a regular diet (NC group), mice in the HFD group presented observably lower heat production (kcal/h/kg), RER, VO_2_ (ml/h/kg) and VCO_2_ (ml/h/kg) (Figures 2B–E), indicating a decreased aerobic metabolic rate. Administration of CLA elevated the VO_2_, VCO_2_ and heat production (Figure 2B, 2D,E), meaning that CLA prevented an HFD-induced decrease in the aerobic metabolic rate. Due to the unbalanced mass gain between the WAT and BAT (Figure 1), it is feasible to attribute the HFD-induced decrease of the metabolic rate to the excessively accumulated visceral and subcutaneous white fat, and the CLA-increased metabolic rate to the reduced fat mass gain. In this experiment, CLA did not affect the RER of mice, and all the three groups of mice showed inseparable XAMB curves.

It is notable that, during the 48 h monitoring period, mice in the CLA group had significantly reduced total energy intake (*p* < 0.05) (Figure 2A) compared to mice in the HFD group, directly contributing to the reduced body weight gain and fat accumulation. These results strongly suggest that CLA plays a role in the regulation of feeding behavior.

### CLA Down-Regulated the Expression of Appetite-Promoting Genes in the Hypothalamus

**FIGURE 3:**
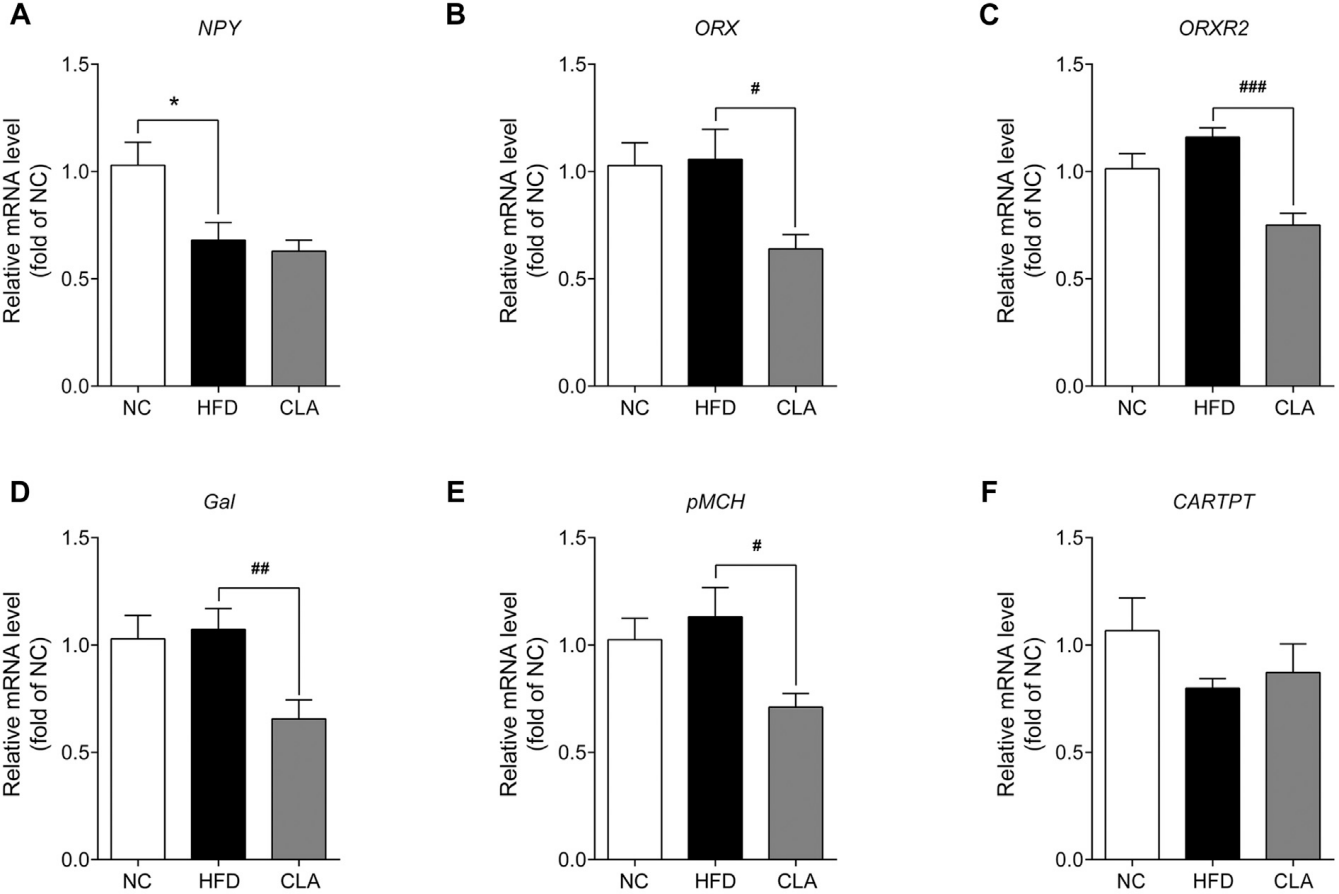
Effect of CLA on the appetite-related genes in the hypothalamus of mice given a HFD. Mice were sacrificed, and their hypothalamic were isolated at the end of the experiment for RT-qPCR analysis of the appetite-related genes. **(A**–**F)** Hypothalamic mRNA levels of *NPY*, *ORX*, *ORXR2*, *Gal*, *pMCH*, and *CARTPT* relative to *β-actin* were measured by qPCR. Data are shown as the mean ± SEM (*n* = 6). *p < 0.05 vs. NC. ^*#*^ *p* < 0.05, ^*##*^ *p* < 0.01, ^*###*^ *p* < 0.001 vs. HFD.

As CLA reduced the energy intake of the mice fed a HFD, the effects of CLA on the mRNA expression of appetite-promoting genes (*NPY*, *ORX*, *ORXR2*, *pMCH*, and *Gal*) as well as the appetite-suppressing gene (*CARTPT*) in the hypothalamus were then detected. The results showed that HFD induced downregulation in the expression of *NPY* without affecting the expression of the other five genes, while intervention with CLA could not reverse the HFD-induced downregulation of *NPY* or affect the expression of *CARTPT* but did significantly down-regulated the expression of *ORX*, *ORXR2*, *pMCH*, and *Gal* (Figure 3). These results strongly support that the reduction in the energy intake is achieved by suppressing appetite-promoting genes in the hypothalamus.

### HFD or CLA did Not Affect the Thermogenic Activity of the brown Adipose Tissue

**FIGURE 4:**
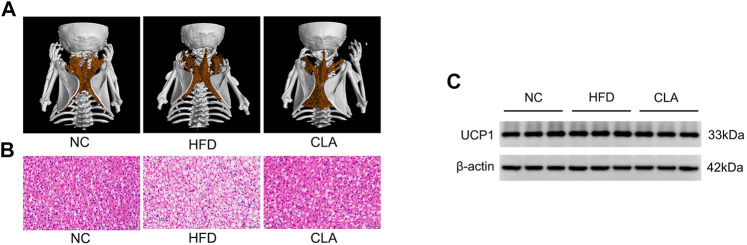
Effect of CLA on the BAT function of mice given a HFD. **(A)** Representative three-dimensional images of BAT reconstructed via micro CT. **(B)** Representative H and E staining of BAT sections. Original magnification, × 400. Scale bar, 20 μm. **(C)** Detection of UCP1 expression in BAT by Western-blotting (*n* = 3 in each group).

Excess fat-induced hypertrophy may impair the BAT function (Villarroya et al., 2017). CLA, shows an ability to protect the heat producing capacity of mice fed a HFD, was then investigated for its effect on BAT activity. In our study, HFD induced fat accumulation within the brown adipocytes, while treatment with CLA effectively prevented HFD-induced fat accumulation within the cells, as expected (Figures 4A,B). Interestingly, however, neither the BAT mass (Figure 1G) nor the BAT expression of UCP1 was affected by HFD and/or CLA (Supplementary Figure 4SC). Based on these results, it is easy to deduce that HFD did not affect the UCP1-dependent thermogenesis of BAT, and CLA did not function by targeting BAT-UCP1. A possible reason for these results is that the amount of fat within the cells was not sufficient to induce cell dysfunction.

### CLA Inhibited HFD-Induced Adipocyte Hypertrophy, UCP1 Suppression and Mitochondrial Damage in the Inguinal White Adipose Tissue

**FIGURE 5:**
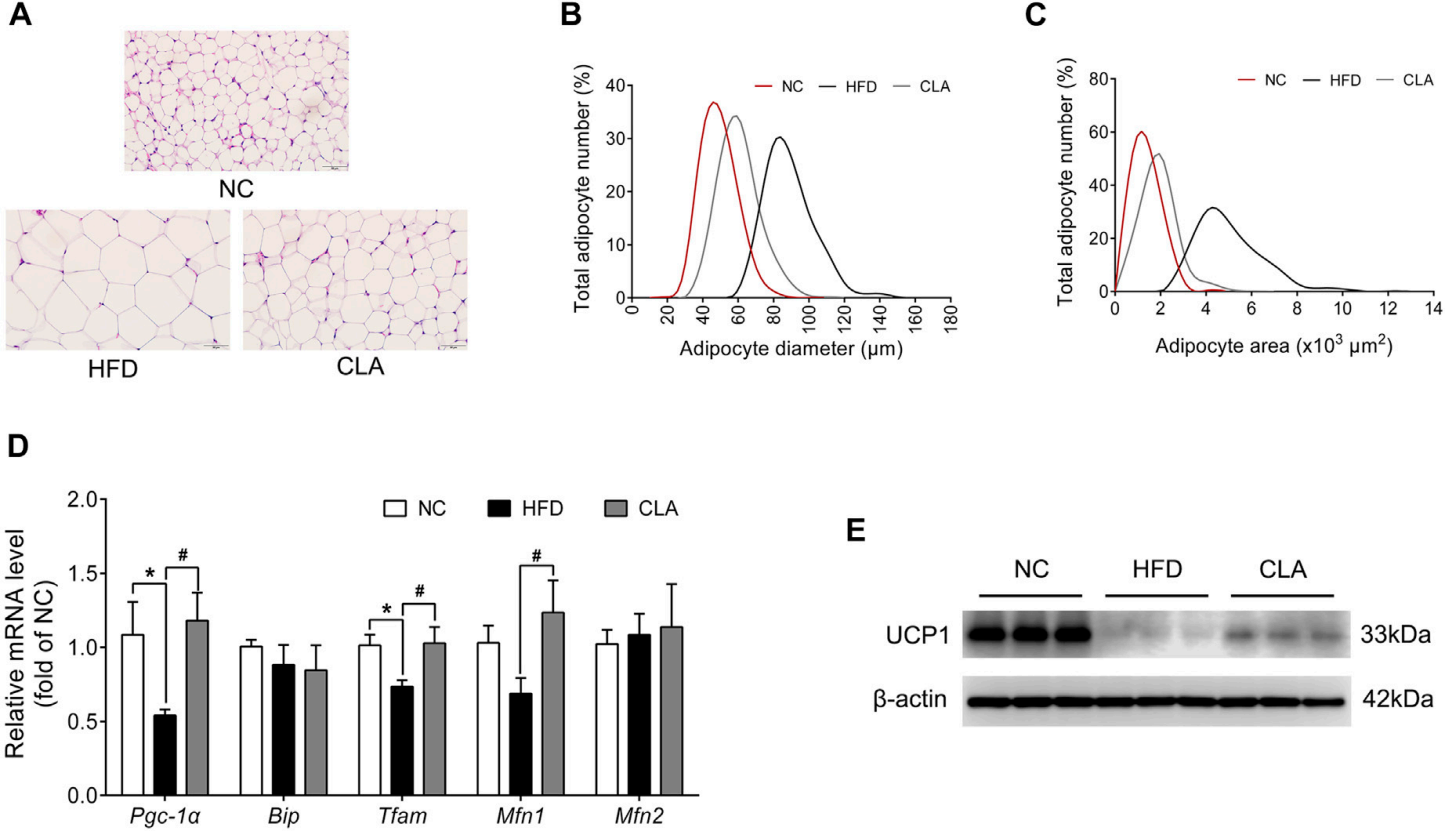
Effect of CLA on the iWAT function of mice fed a HFD. **(A)** Representative H and E staining of iWAT sections. Original magnification, × 400. Scale bar, 50 μm. **(B)** Adipocyte diameter and **(C)** Adipocyte area are shown (*n* = 6). **(D)** mRNA levels of *Pgc-1α*, *Bip*, *Tfam*, *Mfn1*, and *Mfn2* normalized to β*-actin* (*n* = 6). **(E)** Detection of UCP1 by Western blotting (*n* = 3 in each group). *p < 0.05 vs. NC; ^*#*^ *p* < 0.05 vs. HFD.

One of the strategies to fight against obesity is to stimulate the browning of subcutaneous WAT (Kim and Plutzky, 2016). Whether CLA antagonizes HFD-induced suppression to UCP1 and the mitochondrial function of the subcutaneous WAT was then investigated using the iWAT, and the results are shown in Figure 5. In this study, induction with a HFD for 16 w caused adipocyte hypertrophy in the iWAT (Figures 5A–C), as expected, alongside downregulation in the protein level of UCP1 and the mRNA levels of *Pgc-1α* and *Tfam*, which are genes involved in thermogenesis and mitochondrial biogenesis (Figures 5D; Supplementary Figure 5SE), indicating that the HFD resulted in a decrease in the number of beige adipocytes and mitochondria in the subcutaneous adipose tissue. Treatment with CLA effectively alleviated HFD-induced adipocyte hypertrophy in the iWAT (Figures 5A–C), with the mRNA levels of *Pgc-1α*, *Tfam*, and *Mfn1* (mitochondrial function-related gene) restored to normal levels (Figure 5D), and the protein level of UCP1 partially restored (Supplementary Figure 5SE). These results suggest that CLA has protective effects on beige adipocytes and mitochondria in the iWATs of mice fed a HFD.

### CLA Prevented HFD-Induced Adipocyte Hypertrophy, Angiogenesis and Mitochondrial Damage in the Perigonadal White Adipose Tissue

**FIGURE 6:**
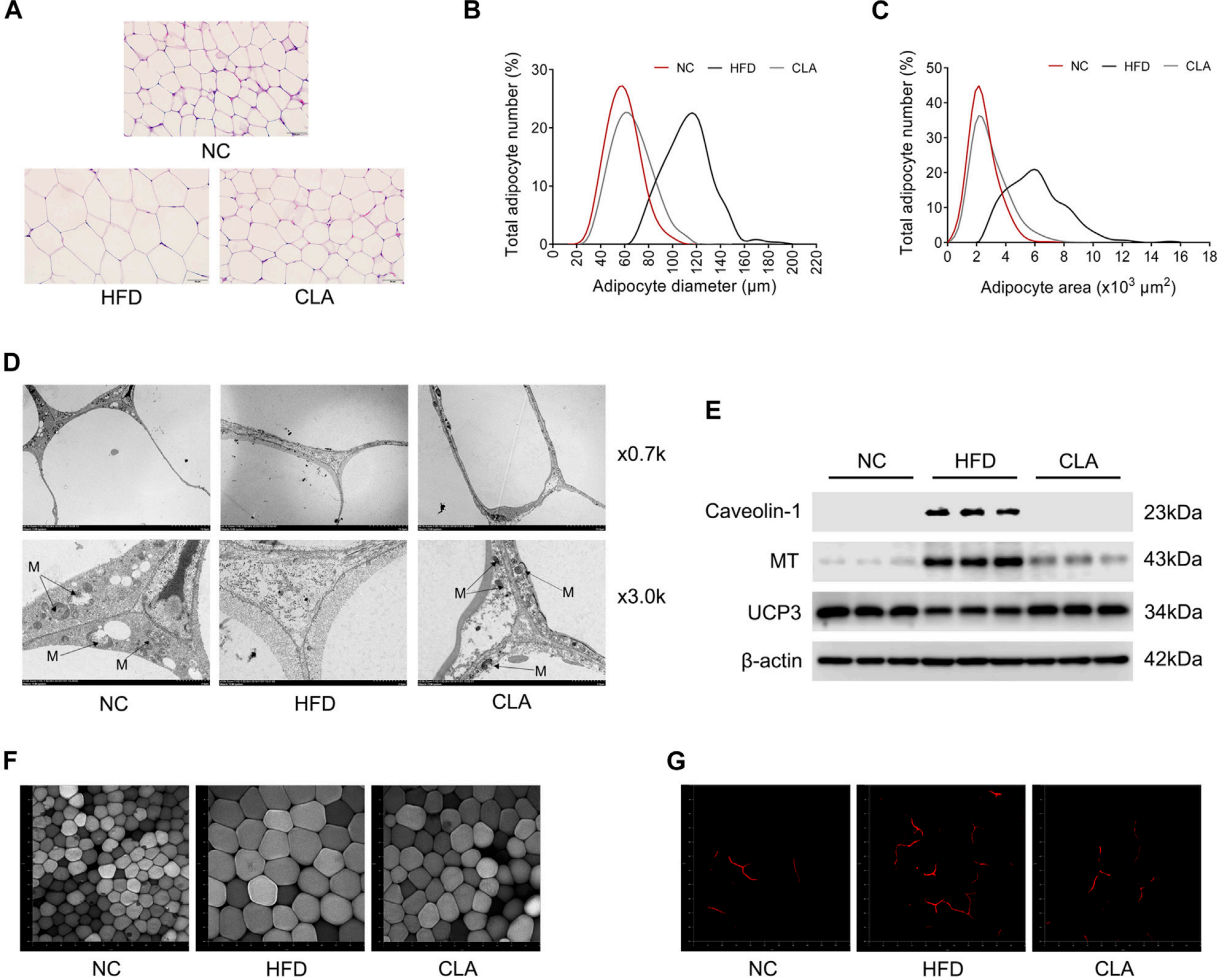
Effect of CLA on the pWAT of mice induced with a HFD for 16 w. **(A)** Representative H and E staining of pWAT sections. Original magnification, × 400. Scale bar, 50 μm. **(B)** Adipocyte diameter and **(C)** Adipocyte area are shown (*n* = 6). **(D)** Representative transmission electronic microscopy images of pWAT. M, mitochondria; original magnification, × 0.7 k and × 3.0 k. Scale bar, 10.0 μm. **(E)** Detection of Caveolin-1, MT and UCP3 by Western blotting (*n* = 3 in each group). **(F)** Representative fluorescence characterization of LipidTOX for detection of the adipocytes. **(G)** Representative fluorescence characterization of PECAM-1 for the detection of angiogenesis. Original magnification, × 200. Scale bar, 50 μm.

Lipid-induced inflammation or oxidative stress impairs the mitochondrial function and energy metabolism of cells, as observed in our previous study (Luo et al., 2020). In this study, the protective effects of CLA on the adipocytes and mitochondria of visceral adipose tissue were investigated using the pWAT, and the results are shown in Figure 6. CLA effectively inhibited the HFD-induced hypertrophy of adipocytes (Figures 6A–C,F); upregulation in the expression of caveolin-1 and malonyl CoA: ACP transacylase (MT) (Figure 6E), which are involved in lipid storage and lipogenesis; downregulation in the expression of thermogenic protein UCP3 (Supplementary Figure 6SE), which is an analogue of UCP1; and decrease in cellular mitochondria (Figure 6D) in the pWAT. In addition, angiogenesis in the pWAT, which is necessary for tissue expansion, was also inhibited by CLA (Figure 6G). These results suggest that CLA is able to protect the visceral adipose tissue from HFD-induced expansion, mitochondrial dysfunction, and reduction in heat production.

### CLA Prevented HFD-Induced Metabolic Disorder in the Liver

**FIGURE 7:**
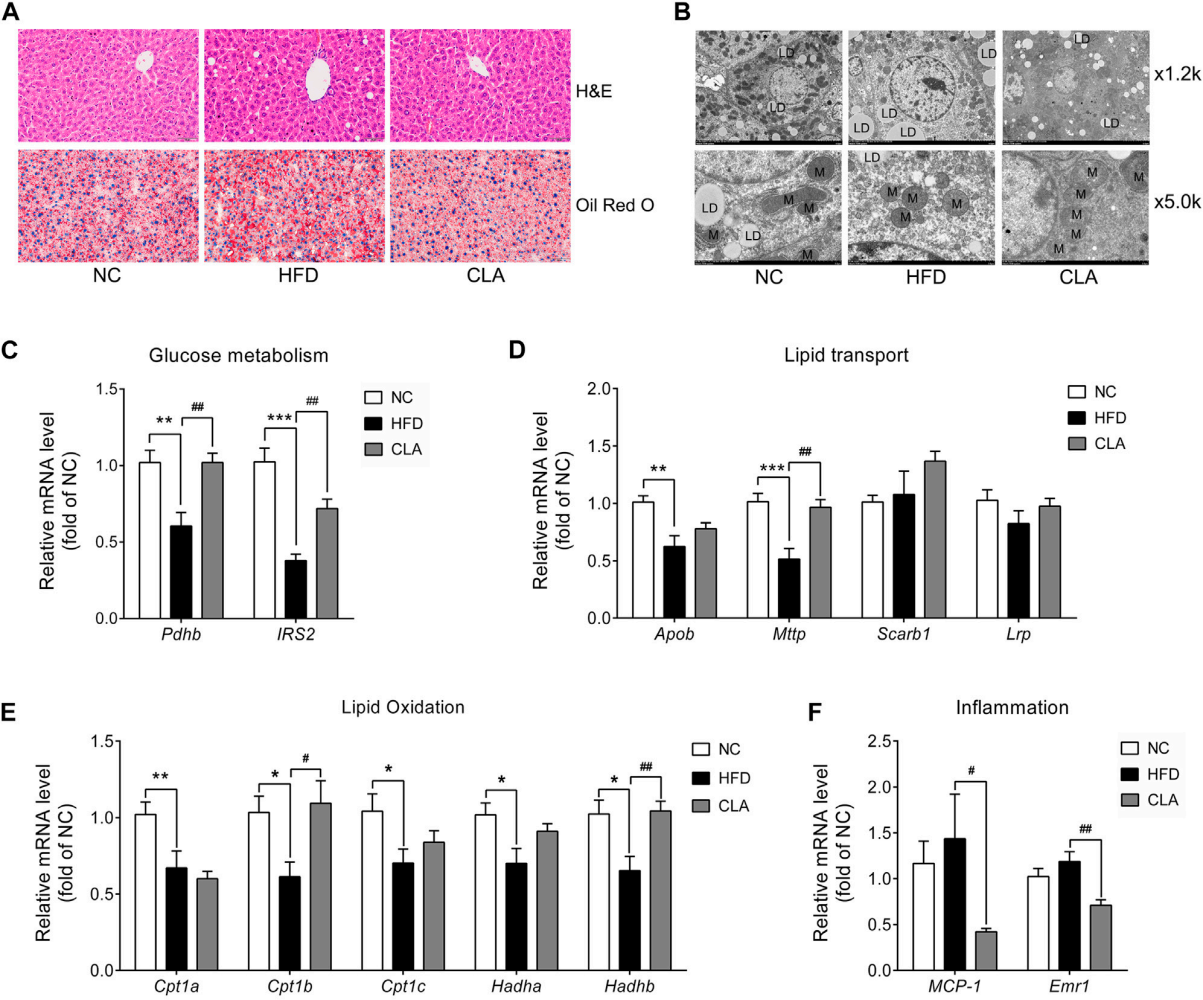
Effects of CLA on the livers of HFD-induced mice. **(A)** Representative H and E staining (above) for histopathology and oil red O staining (below) for the detection of triglycerides (red color) of the liver sections. Original magnification, × 400. Scale bar, 20 μm. **(B)** Representative transmission electronic microscopy images of the liver. L, lipid droplet; M, mitochondria; original magnification, × 1.2 k and × 5.0 k. Scale bar, 10.0 μm. **(C**–**F)** Relative mRNA levels of genes involved in glucose metabolism, lipid transport, lipid *β*-oxidation, and inflammation normalized to *β-actin* (*n* = 7). *p < 0.05, **p < 0.01, ***p < 0.001 vs. NC; ^*#*^ *p* < 0.05, ^*##*^ *p* < 0.01 vs. HFD.

The protective effects of CLA on liver function were investigated, and the results are shown in Figure 7. Compared to mice in the NC group, mice in the HFD group exhibited moderate liver steatosis (Figure 7A), with more lipid droplets and fewer mitochondria within the liver cells (Figure 7B). Moreover, downregulation in the mRNA expression of genes involved in glucose metabolism (*Pdhb* and *IRS2*), lipid output (*Apob* and *Mttp*), and fatty acid *β*-oxidation (*Cpt1a*, *Cpt1b*, *Cpt1c*, *Hadha*, and *Hadhb*) (Figures 7C–E) was observed, indicating impaired insulin sensitivity, lipid output, and mitochondrial function of the liver cells. Compared to livers in the HFD group, livers in the CLA group contained fewer triglycerides (Figures 7A,B) and more mitochondria; up-regulated mRNA levels of *Pdhb*, *IRS2*, *Mttp*, *Cpt1b*, and *Hadhb* (Figures 7C–E); and down-regulated mRNA levels of inflammatory genes *MCP-1* and *Emr1* (Figure 7F). These results suggest that CLA might be able to prevent HFD-induced liver dysfunction.

## Discussion

In this study, CLA effectively prevented HFD-induced weight gain and metabolic disorder in female C57BL/6 mice with normal ovarian function, indicating its application prospects in people, at least in females, either premenopausal or postmenopausal, to prevent high-calorie foods-induced obesity and related metabolic syndrome.

Doubtlessly, it is the reduced energy intake, but not the increased energy expenditure contributes most to the obesity prevention effects of CLA, and most likely, through a mechanism of suppressing the hypothalamic expression of orexigenic genes (Figures 2, 3).

Orexigenic genes in the hypothalamus not only regulate feeding behavior, but also play roles in regulating adipocyte homeostasis, BAT function and energy metabolism by stimulating the receptors and downstream signaling pathways (Goforth and Myers, 2017; Sellayah et al., 2011; Marsh et al., 2002; Kyrkouli et al., 2006). In our studies under high-fat feeding conditions, the hapothalami of mice with ovaries expressed down-regulated level of *NPY* and unchanged levels of *ORX* and *Gal* in the hypothalamus (Figure 3), on the contrary, the hypothalami of mice subjected to ovariectomy expressed up-regulated levels of *ORX* and *Gal* but unchanged level of *NPY* (Luo et al., 2021). Based on the results that CLA suppressed same genes (*ORX*, *ORXR2*, *pMCH*, and *Gal*) in the hypothalamus and reduced energy intake in both mice, we suggest that CLA has the prospect to prevent excess accumulation of fat in both premenopausal and postmenopausal women. However, the modes by which CLA regulates the transcription of these genes are unclear and needed to be investigated.

Beige adipocytes in the subcutaneous adipose tissue and brown adipocytes in the BAT dissipate energy in the same way, but they have different progenitors (Wu et al., 2012; Lynes and Tseng, 2015; Lizcano, 2019). In our study, induction with HFD for 16 w caused a significant downregulation in the expression of UCP1 in the iWAT but not in the BAT of mice, suggesting that HFD preferentially impairs the differentiation and formation of beige adipocytes and thus the heat production by subcutaneous adipose tissue. Treatment with CLA did not affect the UCP1 level in the BAT or the mass of BAT and only partially reversed the HFD-induced inhibition to UCP1 in the iWAT, suggesting that CLA does not work by stimulating the expression of UCP1 but instead by protecting the beige adipocytes from HFD-induced damage.

Caveolin-1 modulates both lipid droplet biogenesis and metabolism and is necessary for efficient lipid droplet formation (Fernández et al., 2006), while MT is a mitochondrial enzyme that participates in the synthesis of palmitic acid and triglycerides (Cohen and Fisher, 2013). In our study, the overexpression of caveolin-1 and MT was observed in the pWATs of the HFD group, but with the treatment of CLA, the overexpression of caveolin-1 and MT was inhibited, meaning that CLA prevented HFD-induced lipid droplet formation and lipogenesis in visceral white adipocytes. Moreover, CLA prevented HFD-induced downregulation in the expression of UCP3, whose abundance is closely related to fatty acid β-oxidation (Pohl et al., 2019), showing the potential to protect the visceral adipose from HFD-induced fat accumulation.

HFD is commonly used to induce non-alcoholic fatty liver disease (NAFLD) in animals (Pan and An, 2018; Lv et al., 2019). Hepatocytes, which control the synthesis, storage, transport and metabolism of triglycerides (Vasconcellos et al., 2016), become dysfunctional in fatty liver. In our study, HFD induced downregulation in the mRNA expression of Apolipoprotein B (*Apob*) and microsomal triglyceride transfer protein (*Mttp*) which are responsible for the output of triglycerides from hepatocytes (Berriot-Varoqueaux et al., 2000; Schonfeld, 2003), and of carnitine palmitoyltransferase1 (*Cpt1*), which participates in fatty acid *β*-oxidation (Savage et al., 2006), meaning that long-term HFD impairs lipid output and metabolism in the liver. CLA antagonized HFD-induced suppression in the mRNA expression of *Cpt1b* and *Mttp*, and prevented the liver dysfunction, highlighting its potential to fight against NAFLD.

Mitochondrial dysfunction contributes to the development of insulin resistance and other metabolic diseases (Lee et al., 2019). CLA showed protective effects on the mitochondrial functions of the adipocytes and hepatocytes, may have the potential to improve glucose/lipid metabolism.

## Conclusion

In our study, CLA protected the female C57BL/6 mice from high-fat diet-induced obesity, liver dysfunction and adipose tissue dysfunction, showing the potential to prevent diet-induced weight gain. The underlying mechanism involves the suppression of orexigenic genes in the hypothalamus, which prevents excess energy intake and the subsequent fat accumulation within the adipose tissues and liver and, consequently, lipid-induced tissue dysfunctions.

## Data Availability

The original contributions presented in the study are included in the article/Supplementary Material, further inquiries can be directed to the corresponding authors.
