# Supplementary figures and images for: Cajanolactone A, a Stilbenoid From Cajanus canjan (L.) Millsp, Prevents High-Fat Diet-Induced Obesity via Suppressing Energy Intake
Source: Front Pharmacol. 2021 May 31;12:695561. doi: 10.3389/fphar.2021.695561 (PMC8201603; doi:10.3389/fphar.2021.695561)

## Original scans of immunoblots.

**Figure 4c**

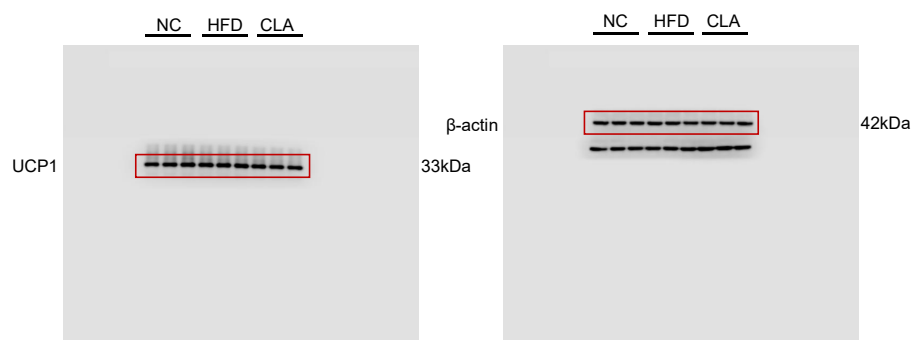

**Figure 5e**

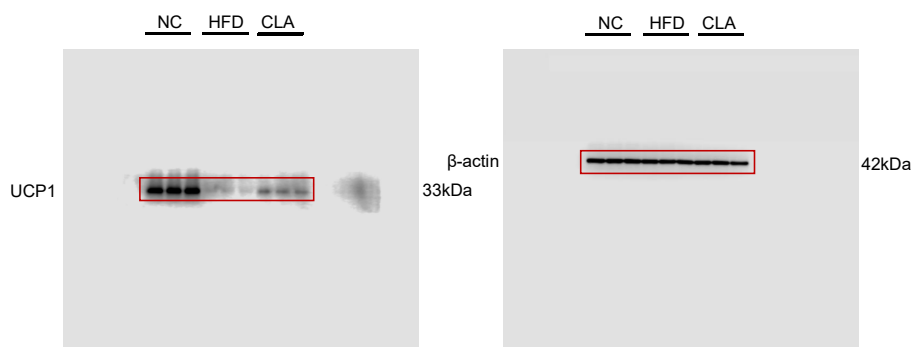

**Figure 6e**

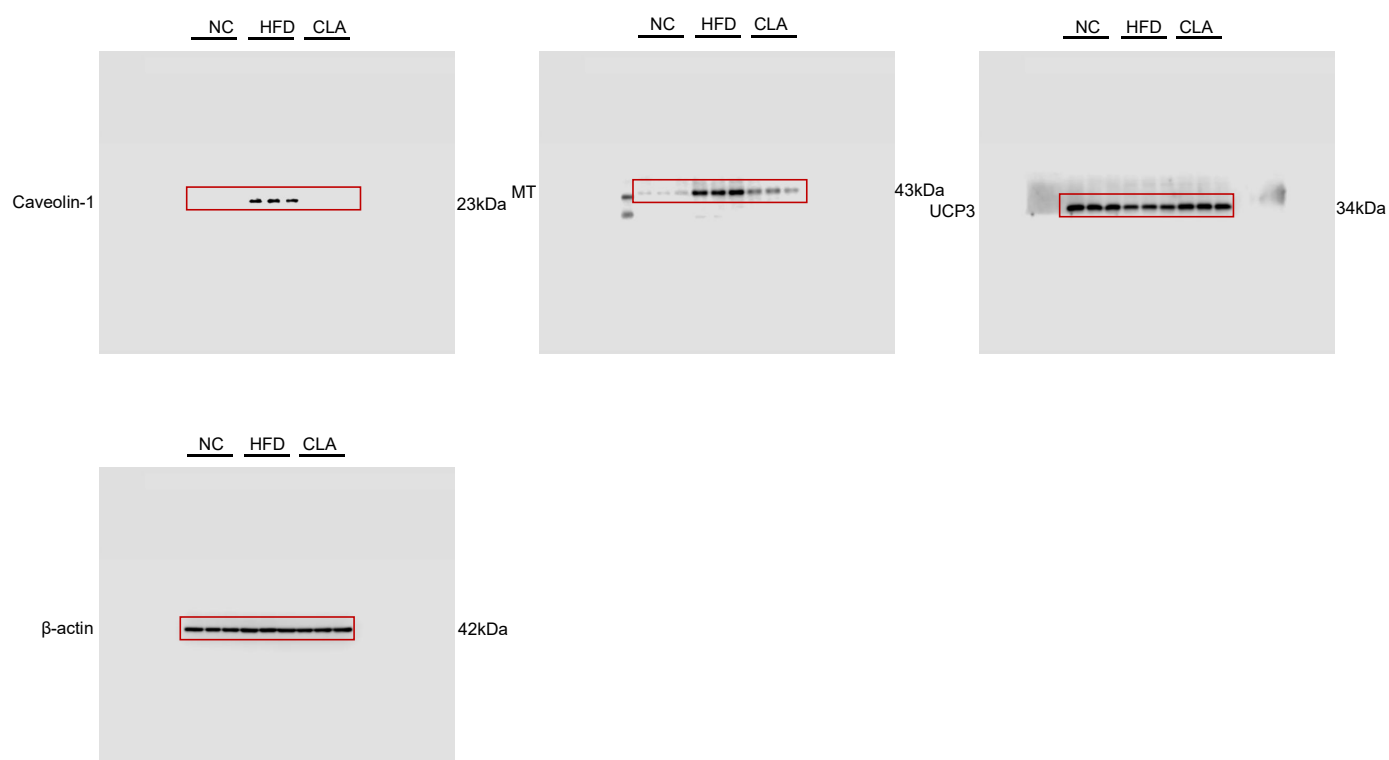

Supplement: Supplementary file 1 [file DataSheet1.PDF]
